# Progress in Medicine

**Published:** 1902-10-25

**Authors:** 


### Progress in Medicine.

(*Continued from page* 49.)

**Typhoid Fever.**—Professor Corfield[44] in the Milroy Lectures deals exhaustively with the etiology of typhoid fever. Reviewing the history of the disease as found in medical literature, he shows that more than a century ago many famous observers distinguished typhoid from typhus fever, although most British physicians regarded them as identical until Jenner taught they were "essentially distinct diseases" in 1849. By copious references to reports of many carefully investigated epidemics at home and abroad he shows the correctness of the current views as to its propagation. Polluted water pre-eminently, milk, and in lesser degree some other articles of food as oysters and other shell fish, ice cream, and uncooked vegetables are responsible for its spread, but entrance of foul air from sewers into houses, dust, and perhaps flies also contribute. Also "communication by direct contagion is a much more common cause of spread of typhoid fever than is generally supposed." The behaviour of the bacillus of Eberth in milk and in various soils is also touched on, and the work of Sidney Martin and others quoted in this connection. Martin found the bacillus flourished best in cultivated soils, especially in gardens and around houses. The late Sir G. Buchanan showed that the death-rate from typhoid fever was specially lowered in towns where refuse matters were quickly removed. Defective sewerage and drainage have contributed, in the opinion of the medical officer of health,[45] to the recurrence of epidemic typhoid in Folkestone for several years—infected milk being the primary cause. Jameson[46] reports an outbreak in Melbourne, also due to infected milk primarily. Later several cases appeared traceable to infection from the first case two months after his discharge, "cured," from hospital. Buesing[47] mentions a soldier who contracted typhoid at Tientsin in October 1901. Four months after his discharge as recovered, in April 1902, typhoid bacilli were present in the urine, but disappeared immediately on administration of urotropin, 2½ grams daily. J. J. L[illegible]mann[48] remarks on, and illustrates by cases, the difficulty of in some cases distinguishing malaria and typhoid. Both may be present. In his cases Widal's reaction and the plasmodium malariæ were obtained erratically. He quotes Osler, who says, "regard every case of continued fever as guilty, that is as typhoid, until the contrary be clearly demonstrated." Similar confusion may sometimes arise with regard to typhoid and trichinosis, although the actual conjunction of these two diseases in one patient is rare. Fever, general malaise, and intestinal features are common to both, and in addition rose spots, enlarged spleen, and the diazo reaction in the urine may occur. T. McCrae[49] reports a case of double infection which occurred in Dr. Osler's clinic. The patient, a German, aged 23, was accustomed to eat raw sausage. Clinically he presented all the features of typhoid fever with, in addition, some puffiness of the eyes, and tenderness of the calf and cervical muscles. Widal's reaction was obtained. The eosinophilia observed on repeated examination of the blood led to the suspicion of trichinosis, the association of eosinophilia and trichinosis having been first pointed out by Brown in 1897. Examination of a fragment of the gastrocnemius, however, proved negative. Later trichinæ were demonstrated in a fragment of the biceps muscle removed under cocaine. Maude[50] notes a case of uric acid calculi where the blood gave Widal's reaction. The reaction was also obtained in a boy, aged 16, who died of advanced multilobular cirrhosis of the liver.[51] At the autopsy, no lesions of typhoid were found. In both these cases a previous attack of typhoid seemed unlikely. J. N. Henry[52] calls attention to the not very rare occurrence of fæcal impaction, of which diarrhœa may be a symptom. Overdosage with milk may be a contributing factor. Abortion or premature delivery[53] occur frequently in typhoid fever—in two-thirds of all cases where pregnancy and typhoid coexist. The accident is grave occurring in a patient weakened by illness, and there is increased liability to hæmorrhage and septic complications. Typhoid bacilli have been found in the blood and tissues of the fœtus. Mr. Brodrick stated recently in the House of Commons [54] that a scheme had been adopted for providing soldiers with "safe water," and that special instructions had been issued to regimental officers with regard to the necessity of their preventing their men from drinking unfit water. C. Childs [55] describes and figures a "kitchen cart" used in the Swiss army, in which the meal can be cooked during transport. The Russians employ a similar article. He thinks such a cart might be modified to provide boiled water also. Full statistics showing the value of inoculation in South Africa are not yet available. One group published by A. Crombie [56] shows a greater incidence among the inoculated than among the uninoculated, but the figures are open to the correction of a higher case mortality among the uninoculated. J. H. Bellamy [57] gives notes of 95 cases of enteric occurring in the Sheffield Union Infirmary. The mortality was 9·4 per cent. All cases were treated with chlorine water according to Burney Yeo's prescription. Starting with four pints of fluid per diem, mainly milk, the patients were soon advanced to eggs, Benger's food, and plasmon, and were taking bread, fish, and rice pudding before the temperature became normal. Only two cases relapsed.

**Dengue.**—The culpability of the mosquito as an agent of infection appears to be deepened by some experiments carried out by H. Graham,[58] at Beyrouth, in Syria. The town lies at the foot of the Lebanon mountains, surrounded by mulberry groves, and infested by mosquitoes which appear to be all of the genus *culex*. An epidemic of dengue appeared there last summer and spread rapidly. Doubting infection by personal contagion, Dr. Graham isolated several patients under netting, and destroyed the mosquitoes in the houses where these patients were secluded, with the result that nobody else in the houses became infected. He next allowed willing victims to be bitten by mosquitoes fed on dengue patients, and found they developed the disease. Two of the subjects were living in a high mountainous village where no case had occurred. On examining the blood of dengue patients he found in all cases on careful seeking, a protozoon present in the red corpuscles which is described and figured. It somewhat resembles the plasmodium malariæ, but the cycle of changes it undergoes is much slower.

[44] Lancet, March 22, April 5, 12, 26, May 3. [45] Brit. Med. Jour., April 26, p. 1036. [46] Austral. Med. Gaz., April 21. [47] Therapist, July 13. [48] Med. Rec., June 28. [49] Amer. Jour. Med. Sc., July. [50] Brit. Med. Jour., June 7, p. 1401. [51] Lancet, April 26, p. 1179. [52] Amer. Jour. Med. Sci., July. [53] Lancet, May 10, p. 1334. [54] Brit. Med. Jour. May 10, p. 1167. [55] Ibid., March 15. [56] Lancet, May 3. [57] Quart. Med. Jour., May. [58] Med. Rec., Feb. 8, March 1.

*(To be continued.)*

